# Blue Light Aminolevulinic Acid Photodynamic Therapy Did Not Induce Detectable CPD or 6–4PP Photolesions in Human Dermal Fibroblasts

**DOI:** 10.1002/jbio.70328

**Published:** 2026-08-05

**Authors:** Julia Stolyar, Evan Austin, Olumide M. Arigbede, Jared Jagdeo

**Affiliations:** ^1^ Department of Dermatology State University of New York, Downstate Health Sciences University Brooklyn New York USA; ^2^ Dermatology Services Veterans Affairs New York Harbor Healthcare System – Brooklyn Campus Brooklyn New York USA; ^3^ Department of Epidemiology and Biostatistics State University of New York, Downstate Health Sciences University Brooklyn New York USA; ^4^ Office of the Senior Vice President for Research State University of New York, Downstate Health Sciences University Brooklyn New York USA

**Keywords:** 6–4 photoproducts, aminolevulinic acid, blue light, cyclobutane pyrimidine dimers, fibroblast, photodynamic therapy

## Abstract

Photodynamic therapy (PDT) is an established dermatologic therapeutic modality used to induce apoptosis within targeted tissue. Despite increasing clinical use, the genotoxic safety of blue light (BL) PDT has not yet been established. We examined whether combination BL (417 ± 5 nm) and 5‐aminolevulinic acid (5‐ALA) PDT induces cyclobutane pyrimidine dimers (CPDs) and 6–4 photoproducts (6–4PPs) in human dermal fibroblasts. CRL‐2617 and AG‐13145 fibroblasts were treated with 0, 0.5, or 1 mM 5‐ALA, then irradiated with BL at 10, 30, or 45 J/cm^2^. Unlike robust photolesion formation in the UVB‐irradiated positive controls, no BL PDT experimental condition produced CPD or 6–4PP signal exceeding the empirically validated assay lower limit of quantification (LLOQ; 1.5625 ng/mL). However, sub‐LLOQ DNA damage cannot be excluded. The minimum detectable effect was 2.42 ng/mL, ~1.55‐fold the LLOQ. Linear mixed‐effects sensitivity analysis confirmed assay responsiveness, demonstrating clear separation between UVB and BL PDT signals.

## Graphical abstract

This study demonstrates that combination BL and 5‐ALA PDT does not induce detectable levels of CPD or 6–4PP photolesions in human dermal fibroblasts in vitro. Our findings contribute to the lesion‐specific genotoxic safety profile of blue light photodynamic therapy, an established research topic.

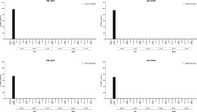

**Abbreviations**
5‐ALA: 5‐Aminolevulinic Acid
6–4PP: 6–4 Photoproducts
BL: Blue Light
CPD: Cyclobutane Pyrimidine Dimers
ELISA: Enzyme‐Linked Immunosorbent Assay
HDF: Human Dermal Fibroblasts
LLOQ: Lower Limit of Quantification
LMM: Linear Mixed‐Effects Model
MAL: Methyl Aminolevulinate
PDT: Photodynamic Therapy
PPIX: Protoporphyrin IX
UV: Ultraviolet

## 1 Introduction

Photodynamic therapy (PDT) is an established noninvasive therapeutic modality for actinic keratosis with growing evidence of its efficacy across a variety of dermatologic conditions, including acne, nonmelanoma skin cancers, photoaging, and alopecia [1, 2, 3, 4]. The global market revenue for PDT devices was estimated at $608 million in 2024, with a projected growth exceeding $1 billion by 2030 [5]. Rising patient demand for light‐based therapies underscores the need for further investigation into their safety profile.

PDT involves the activation of a photosensitizer, either 5‐aminolevulinic acid (5‐ALA) or methyl aminolevulinate (MAL), by a light source in the presence of oxygen [1, 6]. 5‐ALA and MAL are then metabolized to protoporphyrin IX (PPIX), which generates reactive oxygen species that induce controlled apoptotic cellular death within targeted tissue [1, 6]. PDT commonly utilizes wavelengths within the visible light spectrum (400–700 nm). Blue light (BL, 400–500 nm) may be particularly effective as a PDT light source, given that PPIX is preferentially absorbed in the Soret band (405 and 415 nm), which lies within the BL spectrum [1]. BL has a minimal penetration depth of ~1 mm, targeting superficial tissue within the epidermis [7].

The clinical safety of PDT has been well established, with adverse effects generally limited to self‐resolving erythema, edema, and discomfort [2, 6]. Several clinical trials have demonstrated the safety of BL PDT in particular using a range of photosensitizers, including 10% ALA, 20% ALA, and MAL, with no significant adverse events reported [8, 9].

Ultraviolet (UV) light is a well‐known inducer of DNA damage, producing cyclobutane pyrimidine dimers (CPDs) and 6–4 photoproducts (6–4PPs) [10, 11, 12]. These photolesions disrupt replication and transcription by generating mismatched base pairings, leading to the development of extremely mutagenic photoproducts with slow rates of repair [10, 11, 12]. Thus, CPDs and 6–4PPs are widely used biomarkers of DNA damage. Our prior in vitro research demonstrated the genotoxic safety of visible BL (417 ± 5 nm), red light (635 ± 5 nm), and near infrared light (830 ± 5 nm) alone, with no evidence of detectable CPD or 6–4PP formation in human dermal fibroblasts (HDFs) [13, 14, 15]. However, there is no conclusive evidence of whether the combination of BL with a photosensitizer, 5‐ALA, induces DNA damage. There are several conflicting studies presenting data both supporting and refuting the formation of DNA damage following BL phototherapy alone and BL PDT [13, 16, 17, 18, 19]. This inconsistency emphasizes the need for further evaluation of the genotoxic safety of BL PDT. This question has important clinical relevance because non‐lesional skin is also exposed during PDT treatment, raising the possibility that the residual healthy skin cells may sustain DNA damage.

To bridge this knowledge gap, we aimed to determine whether BL PDT induces detectable levels of DNA damage in HDFs in the form of CPDs and 6–4PPs. We hypothesized that even in the presence of a photosensitizer, BL would not induce measurable formation of CPDs or 6–4PPs. Formation of these two photolesions was assessed via enzyme‐linked immunosorbent assay (ELISA) and immunoblot.

## 2 Materials and Methods

### 2.1 Materials

AG‐13145 cells were acquired from Coriell Institute (Camden, New Jersey) and CRL‐2617 cells were acquired from the American Type Culture Collection (Manassas, Virginia). 5‐aminolevulinic acid (5‐ALA) was obtained from Sun Pharmaceuticals (Wilmington, MA). Dulbecco's Modified Eagle's Medium (DMEM), phosphate‐buffered saline (PBS), trypsin, and antibiotic‐antimycotic (ABAM) (100×) were all procured from Thermo Fisher Scientific (Waltham, Massachusetts). Fetal bovine serum (FBS) was sourced from Atlanta Biologicals (Flowery Branch, Georgia). Tissue culture dishes (100 mm) were acquired from Corning (Corning, New York). FlexiGene DNA isolation kits were obtained from Qiagen (Germantown, Maryland). OxiSelect UV‐Induced DNA Damage ELISA Combo kits were purchased from Cell Biolabs (San Diego, CA). Odyssey nitrocellulose membranes, intercept PBS blocking buffer, intercept T20 (PBS) Antibody Diluent, and IRDye 800CW Donkey anti‐Mouse IgG Secondary Antibody were sourced from LI‐COR (Lincoln, Nebraska). Tween 20 was obtained from Bio‐rad (Hercules, California). The BLU‐U Blue Light Phototherapy device (417 ± 5 nm) was sourced from Sun Pharmaceuticals (Wilmington, MA).

### 2.2 Cell Culture

Two HDF cell lines, CRL‐2617 and AG‐13145, were selected as initial reductionist dermal cell models based on previous phototherapy experimentation in our laboratory [13, 14, 15]. This enabled methodological consistency and direct comparison with earlier studies evaluating visible light exposure. Fibroblasts were used to assess baseline genotoxic responses, rather than to model the full complexity of cutaneous tissue. Cells were maintained in a humidified incubator kept at 37°C in 5% carbon dioxide. HDFs were cultured using a medium containing 1 g/L DMEM with 10% FBS and 1% antibiotic‐antimycotic. Cells were plated at a density of 500 000 cells per 100 mm dish. All experiments were performed on cells at passage 12 or lower.

### 2.3 ALA Treatment

Twenty four hours after plating, cells were treated with freshly prepared solutions of 0, 0.5, or 1 mM 5‐ALA in serum‐free culture medium. After 5‐ALA treatment, cells were incubated in the humidified incubator at 37°C in 5% carbon dioxide for 30 min prior to BL irradiation. A 30 min incubation was selected based on our prior laboratory work demonstrating sufficient efficacy of 5‐ALA PDT under short incubation periods [20].

### 2.4 Blue Light Irradiation

After 5‐ALA incubation, experimental cell dishes were irradiated with BL at fluences of 10 J/cm^2^ (1000 s at 10 mW/cm^2^), 30 J/cm^2^ (3000 s at 10 mW/cm^2^), or 45 J/cm^2^ (4500 s at 10 mW/cm^2^). Cells were placed 5 cm directly below the surface of the BLU‐U (Sun Pharmaceuticals), which emits 417 ± 5 nm. Each experimental fluence had a corresponding control dish exposed to only ambient light. These ambient light controls were placed on a heating block at 34°C and covered, thus allowing the control to be matched for temperature, exposure time, humidity, and carbon dioxide levels. The light‐protected negative control remained inside the humidified incubator at 37°C in 5% carbon dioxide to minimize light exposure. The positive control was irradiated with UV‐B light (9 mJ/cm^2^) for 4 min, which is known to induce DNA damage in human dermal fibroblasts [13, 14, 15].

### 2.5 DNA Collection

DNA was harvested from HDFs 3 and 24 h after BL irradiation using the Qiagen FlexiGene DNA isolation kit. DNA was extracted following the manufacturer's instructions. Subsequently, DNA concentration and purity were evaluated using the Nanodrop Spectrophotometer (Thermo Fisher Scientific).

### 2.6 Elisa

CPD and 6–4PP formation was detected using the OxiSelect UV‐Induced DNA Damage ELISA Combo kit according to the manufacturer's instructions. Anti‐CPD and anti‐6‐4 pp. primary antibodies were used for detection, followed by incubation with horseradish peroxidase (HRP)‐conjugated secondary antibodies. Absorbance was quantified with a 96‐well plate reader with an absorbance wavelength of 450 nm. Each plate included an 8‐point calibration curve in technical duplicate spanning nominal CPD‐DNA or 6–4PP‐DNA equivalent concentrations of 0, 1.5625, 3.125, 6.25, 12.5, 25, 50, and 100 ng/mL, generated from the manufacturer's reference standard. Sample concentrations were interpolated from the four‐parameter logistic standard curve as implemented in the OxiSelect data analysis template.

### 2.7 Immunoblot

The DNA samples were heated for 10 min at 95°C, then iced for 10 min. 5 μL of denatured samples were blotted onto the nitrocellulose membranes and air dried at room temperature for 1 h. Next, the membranes were incubated in PBS for 5 min, then in Intercept Blocking Buffer for 1 h. Subsequently, the membranes were incubated with a primary antibody dilution (1:1000 mouse anti‐CPD or anti‐6‐4PP in Intercept T20 Antibody Diluent) for 1 h, followed by blocking with PBS in 0.2% Tween 20 four times. The membranes were then incubated with a 1:1000 antibody dilution (IRDye 800CW donkey anti‐mouse IgG secondary antibody in Intercept T20 Antibody Diluent) for 1 h, again followed by blocking with PBS in 0.2% Tween 20 four times. Finally, the membranes were rinsed with PBS and dried. The membranes were scanned and quantified using the LI‐COR Odyssey M system at a fluorescent intensity of 800 nm.

### 2.8 Statistical Analysis

Descriptive analyses were performed in GraphPad Prism (GraphPad Software, La Jolla, CA, USA). The linear mixed‐effects sensitivity analysis and figure preparation were performed in R version 4.5.3 (R Core Team, 2025) [21]. Each ELISA condition was assayed in two cell lines (CRL‐2617 and AG‐13145), and two independent experimental runs were performed within each cell line using cells of distinct passage numbers. Each ELISA well was loaded in technical duplicate and averaged before analysis. The two passages within each cell line were treated as technical replicates of a common biological source; the two cell lines were treated as the unit of biological replication.

Given this framework, the appropriate statistical approach is a descriptive analytical design stabilized to the lower limit of quantification (LLOQ) of the assay. The LLOQ was defined empirically using bioanalytical acceptance criteria adapted from the U.S. Food and Drug Administration Bioanalytical Method Validation Guidance for Industry and the harmonized ICH M10 guideline on Bioanalytical Method Validation and Study Sample Analysis [22, 23]. These criteria include the lowest non‐zero calibration standard that demonstrated, across all eight ELISA plates assayed in this study (*n* = 16 replicate measurements per standard), an inter‐plate coefficient of variation (%CV) at or below 20% and a mean recovery within 80% to 120% of nominal concentration. Standard G of the OxiSelect calibration ladder, with a manufacturer‐specified nominal concentration of 1.5625 ng/mL CPD‐DNA or 6–4PP‐DNA equivalent, met both criteria (observed mean 1.542 ng/mL; %CV 17.58%; and recovery 98.7%) and was therefore designated as the LLOQ for this study. Shown is the mean signal and observed range over the four experimental runs (two cell lines × two passages) for each tested condition. In each ELISA panel, a horizontal reference line indicates the LLOQ. The inferential claim is constrained as follows: under the conditions tested, no condition produced a CPD or 6–4PP signal above the LLOQ. The bounded null approach adapts the equivalence‐testing tradition formalized by Lakens by using the empirically validated LLOQ as the equivalence bound, in lieu of a researcher‐specified effect size, and is recommended for the inference of non‐meaningful effects in small sample studies and is preferred to underpowered null hypothesis testing [24].

As a sensitivity analysis to confirm assay sensitivity within the present design, a linear mixed‐effects model (LMM) was fit on log_10_‐transformed ELISA concentrations using some libraries in R, such as the lme4 package, with Kenward‐Roger denominator degrees of freedom estimated via the lmerTest package [25, 26]. Values below the limit of detection of the assay calibration model (i.e., values reported as zero) were imputed as LLOQ/2 (0.78125 ng/mL) prior to log transformation, following the convention established by Hornung and Reed for left‐censored bioanalytical data assumed to follow a lognormal distribution [27]. The unified model included treatment (BL PDT vs. UVB positive control, with BL PDT as the reference category), cell line (CRL‐2617, AG‐13145) as a fixed effect, and a random intercept for passage embedded within cell line. Also, 95% of profile‐likelihood confidence intervals were determined by likelihood profiling at *α* = 0.05. The main sensitivity test of the fixed effect of treatment was the degree of separation between BL PDT and UVB on the log₁₀ concentration scale, that is, whether the assay would have detected a positive signal had one been present in any BL PDT condition. Absence of within‐BL PDT variance precluded estimation of fluence‐, dose‐, or timepoint‐specific contrasts within the BL PDT factor. Model output is provided in Table S1A. A formal power analysis quantifying the minimum detectable effect (MDE) at *α* = 0.05 and 80% power is provided in Table S1F using three variance anchors derived from the calibration ladder.

## 3 Results and Discussion

### 3.1 Results

The OxiSelect calibration standard ladder demonstrated strong precision and accuracy across the eight ELISA plates assayed in this study. Inter‐plate coefficients of variation for standards between 1.5625 and 100 ng/mL were within 2.33% and 17.58% and mean recoveries were between 98.7% and 104.6% of the manufacturer‐specified nominal concentrations (Table S1A). The empirical LLOQ was the lowest non‐zero calibration point (Standard G, nominal 1.5625 ng/mL) at which the prespecified bioanalytical acceptance criteria (%CV of ≤ 20%; recovery within 80% to 120%) were met.

**FIGURE 1:**
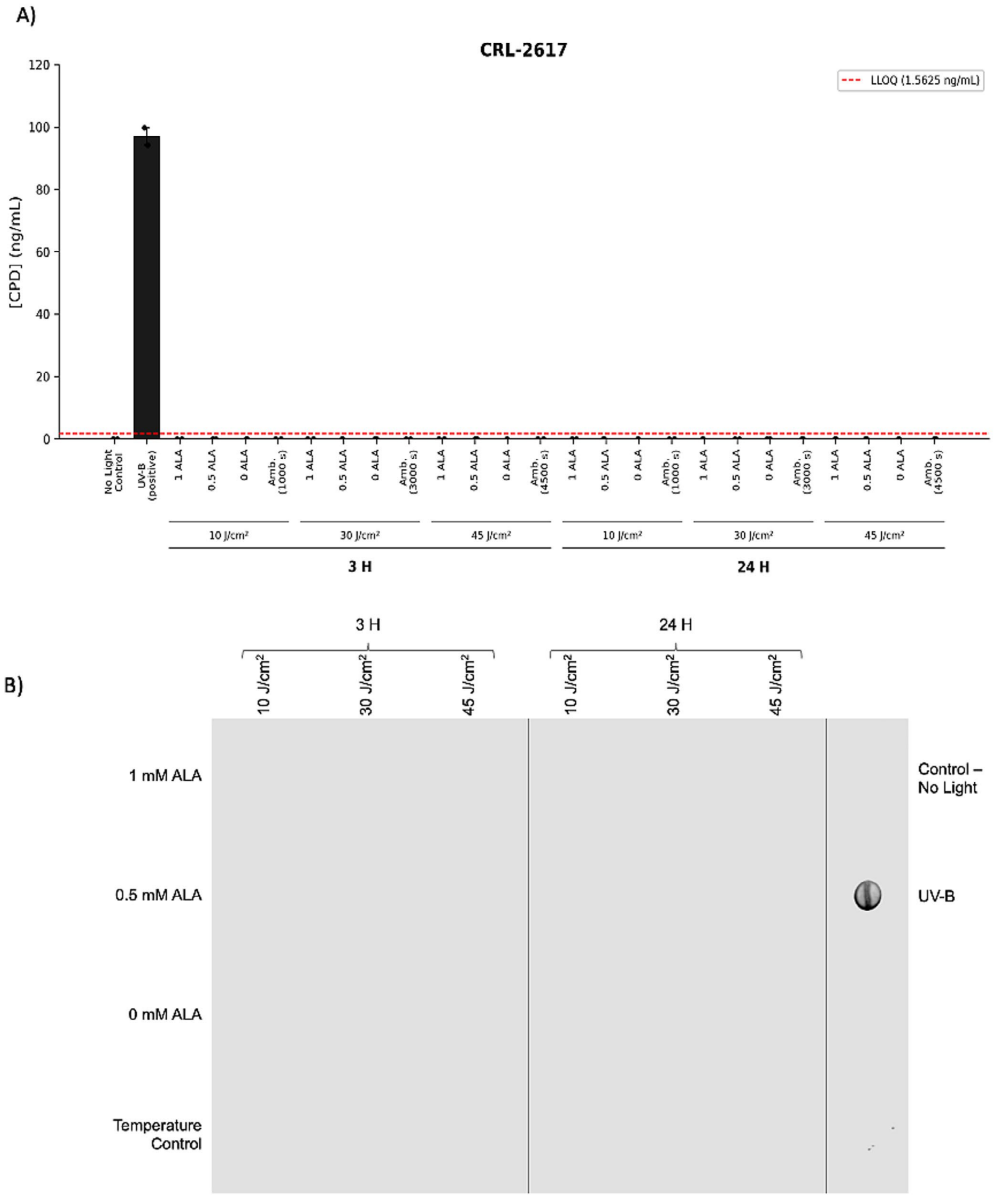
BL PDT did not induce detectable CPD formation in CRL‐2617 HDFs. CRL‐2617 cells were irradiated with 10, 30, 45 J/cm^2^ BL or UVB (9 mJ/cm^2^) following 5‐ALA incubation. DNA was collected 3 and 24 h after irradiation. The presence of CPDs was assessed using (A) ELISA and (B) immunoblot. Each condition was performed in two independent experimental runs (distinct passages); bars represent the mean across runs, and error bars represent the observed range. Individual run values are overlaid as black points. The horizontal red dashed line on panel A indicates the empirically validated assay lower limit of quantification (LLOQ; 1.5625 ng/mL CPD‐DNA equivalent). The UVB positive control is presented as a within‐plate validation of assay sensitivity, not as a tested hypothesis. 1000 s, 1000 s; 3000 s, 3000 s; 4500 s, 4500 s; Amb., temperature‐ and exposure‐time‐matched ambient light control; UVB, ultraviolet B; ALA, aminolevulinic acid; LLOQ, lower limit of quantification.

**FIGURE 2:**
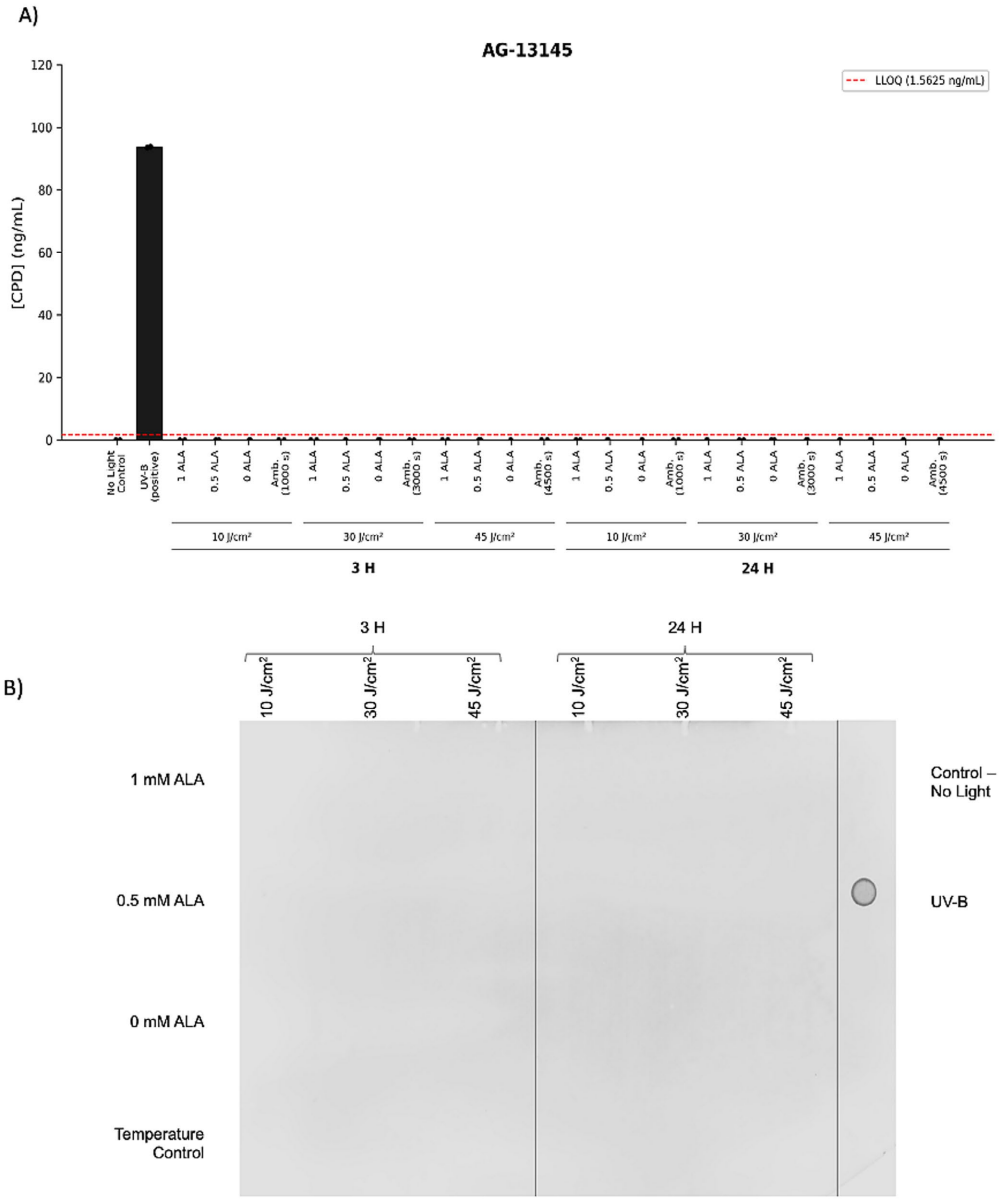
BL PDT did not induce detectable CPD formation in AG‐13145 HDFs. AG‐13145 cells were irradiated with 10, 30, 45 J/cm^2^ BL or UVB (9 mJ/cm^2^) following 5‐ALA incubation. DNA was collected 3 and 24 h after irradiation. The presence of CPDs was assessed using (A) ELISA and (B) immunoblot. Each condition was performed in two independent experimental runs (distinct passages); bars represent the mean across runs, and error bars represent the observed range. Individual run values are overlaid as black points. The horizontal red dashed line on panel A indicates the empirically validated assay lower limit of quantification (LLOQ; 1.5625 ng/mL CPD‐DNA equivalent). The UVB positive control is presented as a within‐plate validation of assay sensitivity, not as a tested hypothesis. 1000 s, 1000 s; 3000 s, 3000 s; 4500 s, 4500 s; Amb., temperature‐ and exposure‐time‐matched ambient light control; UVB, ultraviolet B; ALA, aminolevulinic acid; LLOQ, lower limit of quantification.

**FIGURE 3:**
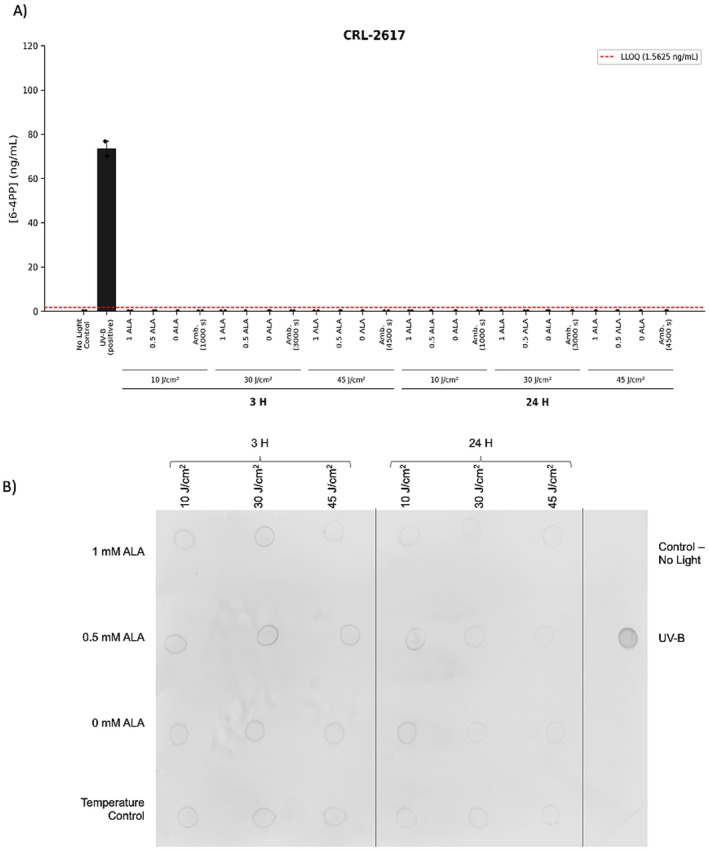
BL PDT did not induce detectable 6–4PP formation in CRL‐2617 HDFs. CRL‐2617 cells were irradiated with 10, 30, 45 J/cm^2^ BL or UVB (9 mJ/cm^2^) following 5‐ALA incubation. DNA was collected 3 and 24 h after irradiation. The presence of 6–4PPs was assessed using (A) ELISA and (B) immunoblot. Each condition was performed in two independent experimental runs (distinct passages); bars represent the mean across runs, and error bars represent the observed range. Individual run values are overlaid as black points. The horizontal red dashed line on panel A indicates the empirically validated assay lower limit of quantification (LLOQ; 1.5625 ng/mL 6–4PP‐DNA equivalent). The UVB positive control is presented as a within‐plate validation of assay sensitivity, not as a tested hypothesis. 1000 s, 1000 s; 3000 s, 3000 s; 4500 s, 4500 s; Amb., temperature‐ and exposure‐time‐matched ambient light control; UVB, ultraviolet B; ALA, aminolevulinic acid; LLOQ, lower limit of quantification.

**FIGURE 4:**
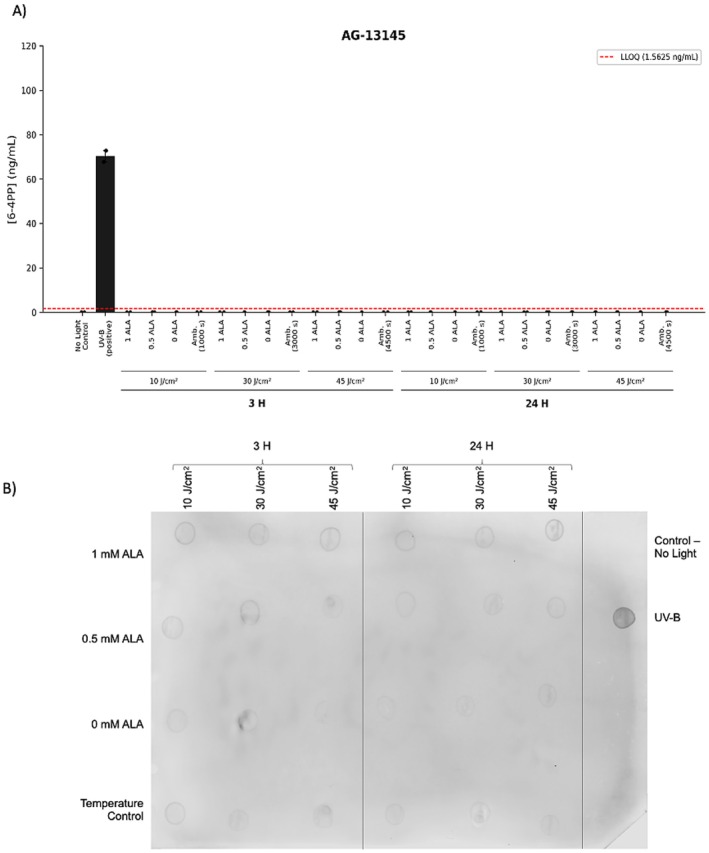
BL PDT did not induce detectable 6–4PP formation in AG‐13145 HDFs. AG‐13145 cells were irradiated with 10, 30, 45 J/cm^2^ BL or UVB (9 mJ/cm^2^) following 5‐ALA incubation. DNA was collected 3 and 24 h after irradiation. The presence of 6–4PPs was assessed using (A) ELISA and (B) immunoblot. Each condition was performed in two independent experimental runs (distinct passages); bars represent the mean across runs, and error bars represent the observed range. Individual run values are overlaid as black points. The horizontal red dashed line on panel A indicates the empirically validated assay lower limit of quantification (LLOQ; 1.5625 ng/mL 6–4PP‐DNA equivalent). The UVB positive control is presented as a within‐plate validation of assay sensitivity, not as a tested hypothesis. 1000 s, 1000 s; 3000 s, 3000 s; 4500 s, 4500 s; Amb., temperature‐ and exposure‐time‐matched ambient light control; UVB, ultraviolet B; ALA, aminolevulinic acid; LLOQ, lower limit of quantification.

Following irradiation of CRL‐2617 and AG‐13145 HDFs with BL at doses of 10, 30, and 45 J/cm^2^ in the presence of 0, 0.5, and 1 mM 5‐ALA, no condition generated CPD or 6–4PP signal exceeding the assay LLOQ at 3 or 24 h after BL irradiation, as assessed by ELISA (Figures 1, 2, 3, 4). For all 192 BL PDT experimental wells (eight plates × 24 BL PDT conditions per plate, including ambient‐light matched temperature controls and light‐protected negative controls), the highest interpolated concentration was 0 ng/mL; no observation was above the LLOQ. The UVB positive control generated a robust photolesion signal that was about 50 to 60‐fold above the LLOQ. This indicates assay sensitivity within each plate (CPD: 93.48 to 99.71 ng/mL across both cell lines; 6–4PP: 67.74 to 76.88 ng/mL).

The linear mixed‐effects sensitivity analysis confirmed assay sensitivity to a known DNA‐damaging agent. The model recovered a fixed effect of UVB versus BL PDT of *β* = 2.086 log_10_ units for CPD (95% profile‐likelihood confidence interval 2.084–2.088 at *α* = 0.05; equivalent to 121.9‐fold separation, 95% CI 121.3–122.6) and *β* = 1.964 log_10_ units for 6–4PP (95% CI 1.959–1.968; 91.9‐fold, 95% CI 91.1–92.8). Cell‐line fixed‐effect estimates did not differ meaningfully between CRL‐2617 and AG‐13145 in either lesion model. Within the BL PDT factor, no within‐condition variance was estimable (all 192 BL PDT observations were at the substitution floor [LLOQ/2 = 0.78125 ng/mL]). The prespecified contrasts of BL fluence, 5‐ALA dose, and timepoint within BL PDT were therefore reduced to point estimates of zero with vanishing standard errors and are not separately interpretable. Thus, the model confirms the descriptive finding that the assay would have detected a positive signal in any BL PDT condition above the LLOQ, and none did.

The ELISA results were confirmed by immunoblot analysis. UVB positive controls showed strong CPD and 6–4PP signal in DNA immunoblot and light‐protected negative controls had baseline signal (Figures 1, 2, 3, 4). Immunoblot signals were at baseline for all BL PDT treatment conditions. Minor variation in background signal intensity was observed in several of the 1 mM 5‐ALA samples (Figure 4B). However, this variation was inconsistent across replicates and not detected by ELISA. Together, these findings indicate that increased doses of 5‐ALA combined with BL exposure did not produce a CPD or 6–4PP signal above the LLOQ of the assay at the in vitro conditions tested.

### 3.2 Discussion

Our study did not demonstrate any detectable photolesional DNA damage in the form of CPDs or 6–4PPs above the assay LLOQ following BL PDT with 5‐ALA treatment in HDFs. No notable CPD or 6–4PP signals were established at BL doses of 10, 30, and 45 J/cm^2^ or at 5‐ALA concentrations of 0, 0.5, or 1 mM. Across two distinct cell lines, CRL‐2617 and AG‐13145, we did not observe any detectable formation of these photolesions at 3 h or 24 h post irradiation. These findings align with our earlier data that BL photobiomodulation, which does not employ a photosensitizer, similarly does not induce detectable formation of CPDs or 6–4PPs [13]. However, the absence of signal above the LLOQ should not be interpreted as evidence of zero DNA damage, absence of biological effect, or broad genotoxic safety.

Earlier studies assessing BL phototherapy alone have achieved opposing conclusions regarding potential genotoxicity. One study reported that exposure of human skin keratinocytes to 4.8 and 9.6 J/cm^2^ BL alone resulted in dose‐dependent generation of oxidative damage and CPDs, respectively, as measured by a comet assay [17]. However, comet assays lack lesion specificity, often yield imprecise measurements, and are influenced by experimental conditions [28, 29, 30]. In contrast, a study investigating B16F1 melanoma cells determined that the lowest dose to produce statistically significant levels of DNA strand breaks occurred after 24 h of 10 W/m^2^ BL irradiation [16]. This corresponds to a fluence of 86.4 J/cm^2^, which is nearly double our highest fluence and far greater than parameters typically used in clinic. Notably, this study demonstrated no detectable CPD formation at any BL dose tested [16].

Furthermore, several studies have also examined whether incubation with 5‐ALA prior to BL exposure affects genotoxic outcomes. An investigation in murine melanoma cells and human‐derived keratinocytes determined that combination BL and 5‐ALA PDT induced DNA double and single‐strand breaks, as well as oxidative damage [18]. Yet, DNA strand breaks and other oxidative lesions are formed by reactive oxygen species, and are thus considered indirect markers of DNA damage [31]. This study also utilized a comet assay, which further limits the specificity of their findings [28, 29]. In contrast, we directly quantified the generation of CPDs and 6–4PPs, the most mutagenic photolesions [10, 11, 12]. Our conclusions are further supported by an in vivo trial in chronically photodamaged facial skin, which demonstrated that broad‐area short incubation BL and ALA PDT did not induce oxidative DNA damage [19]. To our knowledge, no prior study has directly measured CPD and/or 6–4PP formation following combination BL and 5‐ALA PDT treatment. Our findings suggest that no detectable CPD or 6–4PP photolesions were observed following BL PDT at clinically relevant BL fluences and biologically relevant 5‐ALA concentrations, within a controlled in vitro system [20].

One strength of this study is our evaluation of BL exposure in combination with multiple doses of 5‐ALA to determine whether there is a dose‐dependent effect on detectable formation of CPD and 6–4PP photolesions. Furthermore, we used a commercially available BL phototherapy device at fluences that fall within and above standard clinical BL PDT parameters to assess detectable photolesion formation [1]. We also collected data at two time points and compared our findings with positive, negative, and ambient‐light matched controls to counteract potential environmental impacts. Finally, our use of two individual commercially available HDF cell lines enhances reproducibility.

There are also several limitations to consider when interpreting our findings. First, this experiment was performed in vitro using a monolayer of fibroblasts, which does not reflect the multifaceted nature of intact human skin. The use of serum‐free medium during 5‐ALA incubation also represents an in vitro experimental condition and similarly cannot replicate clinical topical delivery of 5‐ALA, because the stratum corneum limits 5‐ALA penetration and PP‐IX accumulation within tissue [32]. Another limitation is that we only evaluated one type of skin cell, fibroblasts. Importantly, fibroblasts are not the primary epidermal targets of PDT. Therefore, this model fundamentally limits extrapolation to keratinocytes, melanocytes, intact epidermis, or clinical treatment settings, which may respond differently upon exposure to BL and 5‐ALA PDT and should also be examined. Because keratinocytes, melanocytes, and other epidermal cells differ from fibroblasts in 5‐ALA metabolism, DNA repair capacity, and susceptibility to photodamage, our findings should not be extrapolated to any other cell types or to intact human skin. Moreover, it is possible that cells taken from more photosensitive individuals may respond differently than the cell lines examined in this study. Additionally, given the achieved sample (two cell lines, two passages per cell line, four total experimental runs per condition), the MDE at *α* = 0.05 and 80% power, calculated using the pooled standard deviation of *σ* = 1.22 ng/mL across the seven non‐blank calibration standards (Table S1F), is 2.42 ng/mL CPD‐DNA or 6–4PP‐DNA equivalent, ~1.55‐fold the empirically validated LLOQ of 1.5625 ng/mL. Consequently, the present study can support claims about CPD and 6–4PP formation only above this quantitative bound; sub‐LLOQ DNA damage cannot be excluded under the tested conditions. Sensitivity analyses using the pooled SD across the lower‐range standards (≤ 12.5 ng/mL; *σ* = 0.46 ng/mL) and the SD at the LLOQ itself (*σ* = 0.27 ng/mL) yield smaller MDE values of 0.92 and 0.54 ng/mL respectively, both below the LLOQ; these anchors describe assay precision at the lower end of the dynamic range and are reported in Table S1F for full transparency. Accordingly, our findings should be considered observational, and the absence of CPD or 6–4PP signal above the LLOQ should not be interpreted as definitive evidence that BL PDT does not induce DNA damage. The multifactorial design of the study (two cell lines, three BL fluences, three 5‐ALA concentrations, two timepoints, two lesion types, two detection methods) was not accompanied by a sample size adequate for fully powered multifactorial inference. We therefore relied on a descriptive primary analysis and a linear mixed‐effects sensitivity analysis as described in Methods. Future studies should use larger numbers of independent biological replicates and a prespecified multifactorial analysis with appropriate correction for multiplicity. Furthermore, we only investigated two forms of photolesional DNA damage, CPDs and 6–4PPs. Though these represent the most mutagenic photolesions, future investigations should analyze other forms of DNA injury, including oxidative DNA lesions like 8‐oxo‐7,8‐dihydroguanine, single‐ and double‐strand breaks, and Dewar valence isomers [10, 33]. Although the UVB positive control confirmed assay response to a known DNA‐damaging agent, we did not perform the combined 5‐ALA + UVB control. Three features of the experimental pipeline materially reduce the likelihood of PPIX‐mediated assay interference. First, the ELISA and immunoblot were performed on extracted, heat‐denatured genomic DNA rather than intact cells, and PPIX, which is predominantly localized to mitochondrial and cytoplasmic membranes, is not expected to co‐purify with double‐stranded DNA through the FlexiGene extraction protocol or to survive heat denaturation. Second, the 450 nm HRP readout falls in a relative absorbance trough of the PPIX spectrum, which has its Soret peak near 405 nm and Q bands centered at ~506, 532, 580, and 630 nm; trace PPIX co‐eluting with the DNA sample would contribute minimally to absorbance at the readout wavelength [1]. Third, the existing UVB positive control validates assay sensitivity to a known DNA‐damaging agent, which is the function it is intended to serve, and the linear mixed‐effects sensitivity analysis quantitatively confirmed this sensitivity (CPD: 121.9‐fold UVB versus BL PDT separation, 95% CI 121.3 to 122.6; 6–4PP: 91.9‐fold, 95% CI 91.1 to 92.8). We acknowledge, nonetheless, that a combined 5‐ALA + UVB experimental arm would provide direct empirical exclusion of PPIX‐mediated interference, and therefore, the possibility of false‐negative results cannot be definitively excluded. Lastly, our BL fluences, 5‐ALA concentrations, and 5‐ALA incubation timing were selected based on our earlier data and clinically relevant parameters; however, alternative technical strategies may yield different results [13, 20].

Future prospectively powered mechanistic studies can build on our findings by exploring additional skin cell types, PDT parameters, markers of DNA damage, and experimental controls to definitively characterize the genotoxic profile of BL PDT. Given the limitations of the present study, our findings should be interpreted as a bounded observation rather than as evidence of broader genotoxic safety. The data support the statement that, under the tested in vitro fibroblast conditions, BL PDT did not generate CPD or 6–4PP signal above the LLOQ of the OxiSelect assay (1.5625 ng/mL DNA damage equivalent). They do not support a general claim of genotoxic safety, do not exclude sub‐LLOQ DNA damage, and do not extrapolate to keratinocytes, melanocytes, intact human skin, or any other cellular context not directly evaluated. Moreover, translating these results to in vivo and ex vivo experiments as well as clinical trials would allow better understanding of BL PDT safety in living human tissue. Expanding on these results to include other wavelengths within the visible light spectrum would further broaden knowledge of the overall safety profile of PDT.

## 4 Conclusion

With the growing popularity of phototherapy, including the implementation of BL PDT into standard dermatologic practice, understanding its molecular safety is becoming increasingly important. Under the specific tested in vitro conditions, this study found that BL 5‐ALA PDT was not associated with CPD or 6–4PP signal exceeding the assay LLOQ in HDFs, while the linear mixed‐effects sensitivity analysis confirmed that the assay would have detected such a signal had it been present. These observations are bounded by the assay LLOQ, the achieved sample size, and the cell type evaluated and should not be interpreted as evidence of overall genotoxic safety of BL PDT. Accordingly, the absence of signal above the LLOQ should be understood as an assay‐bounded observation, not as proof that BL PDT produces no DNA damage or no biological effect. Additional research, including prospectively powered mechanistic, in vivo, ex vivo, and clinical studies, is necessary to definitively establish whether BL PDT induces DNA damage.

## Funding

This study was funded using university start‐up funds from SUNY Downstate and was not sponsored by any pharmaceutical company or government agency.

## Conflicts of Interest

Dr. J.J. has served as a speaker and scientific advisory board member for Sun Pharmaceuticals' educational content and has previously received grants for photodynamic therapy from Sun Pharmaceuticals. Dr. J.J. is also a member of GlobalMed Technologies' scientific advisory board. The remaining authors have no conflicts of interest.

## Supporting information


**Table S1A:** Inter‐plate performance of the OxiSelect calibration ladder.
**Table S1B:** UVB positive control concentrations by cell line and lesion.
**Table S1C:** BL PDT experimental and ambient‐control conditions.
**Table S1D:** Fixed effect estimates from the linear mixed‐effects model.
**Table S1E:** Random effect variance components.
**Table S1F:** Power analysis and minimum detectable effect at α = 0.05 and 80% power.

## Data Availability

The data that support the findings of this study are available from the corresponding author upon reasonable request.
