# Supplementary material for: Blue Light Aminolevulinic Acid Photodynamic Therapy Did Not Induce Detectable CPD or 6–4PP Photolesions in Human Dermal Fibroblasts
Source: J Biophotonics. 2026 Aug 5;19(8):e70328. doi: 10.1002/jbio.70328 (PMC13440324; doi:10.1002/jbio.70328)
Supplement: Supplementary file 1 [file JBIO-19-e70328-s001.docx]

**Supplementary Table S1**

*Manuscript ID 7752508 | Journal of Biophotonics*

**Supplementary Table S1**

Calibration standard performance, descriptive condition-level summary, and linear mixed-effects sensitivity analysis output.

**Part A. Calibration standard performance across all eight ELISA plates**

**Table S1A. Inter-plate performance of the OxiSelect calibration ladder**

| **Standard** | **Nominal (ng/mL)** | **n** | **Mean observed (ng/mL)** | **SD** | **%CV** | **% recovery** |
| --- | --- | --- | --- | --- | --- | --- |
| A | 100.0 | 16 | 101.528 | 2.369 | 2.33 | 101.5 |
| B | 50.0 | 16 | 50.804 | 1.575 | 3.10 | 101.6 |
| C | 25.0 | 16 | 25.350 | 1.221 | 4.82 | 101.4 |
| D | 12.5 | 16 | 12.487 | 0.689 | 5.52 | 99.9 |
| E | 6.25 | 16 | 6.237 | 0.439 | 7.04 | 99.8 |
| F | 3.125 | 16 | 3.270 | 0.350 | 10.70 | 104.6 |
| G | 1.5625 | 16 | 1.542 | 0.271 | 17.58 | 98.7 |
| H | 0 | 16 | 0.000 | 0.000 | — | — |

*Notes. Each calibration standard was loaded in technical duplicate on each of eight ELISA plates (four cell line × passage runs × two lesion-type plates), yielding n = 16 measurements per standard. %CV is calculated as the standard deviation of the 16 measurements divided by their mean and multiplied by 100. % recovery is calculated as the mean observed concentration divided by the manufacturer-specified nominal concentration and multiplied by 100. Standard G (nominal 1.5625 ng/mL) was the lowest standard satisfying the prespecified bioanalytical acceptance criteria of %CV ≤ 20% and recovery within 80% to 120%, and was therefore established as the empirical lower limit of quantification (LLOQ) for this study. SD, standard deviation; CV, coefficient of variation; LLOQ, lower limit of quantification.*

**Part B. Descriptive condition-level summary of UVB and BL PDT outcomes**

**Table S1B. UVB positive control concentrations by cell line and lesion**

| **Cell line** | **Lesion** | **Range (ng/mL)** | **Mean (ng/mL)** | **n** | **Status vs LLOQ** |
| --- | --- | --- | --- | --- | --- |
| CRL-2617 | CPD | 93.48 – 99.71 | 96.94 | 4 | > LLOQ |
| AG-13145 | CPD | 93.48 – 93.86 | 93.67 | 4 | > LLOQ |
| CRL-2617 | 6-4PP | 70.16 – 76.88 | 73.52 | 4 | > LLOQ |
| AG-13145 | 6-4PP | 67.74 – 72.85 | 70.29 | 4 | > LLOQ |

*Notes. n = 4 indicates 4 measurements per cell line × lesion combination (2 passages × 2 within-plate replicates of the UVB positive control well). All UVB positive control concentrations exceeded the LLOQ by approximately 50- to 60-fold, validating assay sensitivity within the achieved design.*

**Table S1C. BL PDT experimental and ambient-control conditions**

| **Treatment category** | **Total observations** | **Maximum observed (ng/mL)** | **Mean (ng/mL)** | **Status vs LLOQ** |
| --- | --- | --- | --- | --- |
| BL PDT (10, 30, 45 J/cm² × 0, 0.5, 1 mM 5-ALA × 3 h, 24 h) | 192 | 0.000 | 0.000 | < LLOQ |
| Ambient-light–matched temperature controls | included in 192 | 0.000 | 0.000 | < LLOQ |
| Light-protected negative controls (No Light) | 8 | 0.000 | 0.000 | < LLOQ |

*Notes. Across all 192 BL PDT experimental wells (8 ELISA plates × 24 BL PDT conditions per plate, including ambient-light–matched temperature controls), no observation exceeded the LLOQ. The eight light-protected No Light negative controls (one per plate, in technical duplicate) were also at baseline. ALA, aminolevulinic acid; BL, blue light; LLOQ, lower limit of quantification.*

**Part C. Linear mixed-effects sensitivity analysis output**

**Table S1D. Fixed effect estimates from the linear mixed-effects model**

| **Lesion** | **Parameter** | **β (log₁₀ ng/mL)** | **SE** | **95% CI lower** | **95% CI upper** | **p-value** |
| --- | --- | --- | --- | --- | --- | --- |
| CPD | Intercept (BL PDT, AG-13145) | -0.107 | <0.001 | — | — | — |
| CPD | Treatment: UVB vs BL PDT | 2.086 | <0.001 | 2.084 | 2.088 | < 0.001 |
| CPD | Cell line: CRL-2617 vs AG-13145 | 0.000 | <0.001 | — | — | 1.000 |
| 6-4PP | Intercept (BL PDT, AG-13145) | -0.107 | <0.001 | — | — | — |
| 6-4PP | Treatment: UVB vs BL PDT | 1.964 | <0.001 | 1.959 | 1.968 | < 0.001 |
| 6-4PP | Cell line: CRL-2617 vs AG-13145 | 0.000 | <0.001 | — | — | 1.000 |

*Notes.* *LMMs were fit on log₁₀-transformed ELISA concentrations using lme4 with Kenward-Roger denominator degrees of freedom estimated through the lmerTest package in R. Observations below detection (concentration = 0 ng/mL) were replaced by LLOQ/2 = 0.78125 ng/mL before the log transformation. The model consists of treatment (BL PDT, reference; UVB) and cell line (AG-13145, reference; CRL-2617) as fixed effects, and passage nested within cell line as a random intercept. 95% CIs were calculated using likelihood profiling with α = 0.05. The within-BL PDT variance was zero because all 192 BL PDT observations were uniformly at the LLOQ/2 substitution floor. Thus the model quantifies only the magnitude of UVB-vs.-BL PDT separation. Treatment fixed effect, back-transformed to the linear scale. CPD UVB vs BL PDT = 102.086 = 121.9-fold (95% CI 121.3 to 122.6) 6-4PP UVB vs BL PDT = 101.964 = 91.9-fold (95% CI 91.1 to 92.8) β, fixed-effect estimate; CI, confidence interval; LLOQ, lower limit of quantification; LMM, linear mixed-effects model; SE, standard error.*

**Table S1E. Random effect variance components**

| **Lesion** | **Random effect** | **Variance** | **SD** |
| --- | --- | --- | --- |
| CPD | Passage nested within cell line | 0.000 | 0.000 |
| CPD | Residual | 0.000 | 0.000 |
| 6-4PP | Passage nested within cell line | 0.000 | 0.000 |
| 6-4PP | Residual | 0.000 | 0.000 |

*Notes. Both random and residual variance components are estimated as zero, reflecting the uniform substitution-floor value across all 192 BL PDT observations and the tight clustering of UVB positive control values within each cell line. This is a singular-fit pattern that is expected and reported transparently rather than concealed; it does not invalidate the fixed-effect estimate of UVB-versus-BL PDT separation, which is identified by the between-treatment contrast. SD, standard deviation.*

**Table S1F. Power analysis and minimum detectable effect at α = 0.05 and 80% power**

| **Variance anchor** | **Standards (k)** | **n total** | **Pooled SD (ng/mL)** | **MDE (ng/mL)** | **MDE / LLOQ** |
| --- | --- | --- | --- | --- | --- |
| **Principal anchor: full dynamic range (Standards A–G)** | **7** | **112** | **1.222** | **2.42** | **1.55-fold** |
| Sensitivity anchor: lower-range standards (Standards D–G; ≤ 12.5 ng/mL) | 4 | 64 | 0.465 | 0.92 | 0.59-fold |
| Sensitivity anchor: at the LLOQ itself (Standard G; 1.5625 ng/mL) | 1 | 16 | 0.271 | 0.54 | 0.34-fold |

*Notes. The minimum detectable effect (MDE) was calculated for a two-sample comparison at α = 0.05 (two-sided) and 80% power, using the closed-form expression δ = σ × (z₀.₉₇₅ + z₀.₈₀) × √(2/n), with n = 4 experimental runs per condition (two cell lines × two passages within each cell line). The multiplier evaluates to (1.960 + 0.842) × √(2/4) = 2.802 × 0.707 = 1.981. Three variance anchors are reported to provide full transparency on the choice of σ. The principal anchor pools the standard deviation across all seven non-blank calibration standards (n = 16 measurements per standard), reflecting assay variability across the full dynamic range (1.5625 to 100 ng/mL) over which BL PDT signal, if present, could fall. Two sensitivity anchors restrict σ to the lower-range standards (Standards D through G; concentrations ≤ 12.5 ng/mL) and to the LLOQ itself (Standard G; 1.5625 ng/mL); these anchors describe assay precision at the lower end of the dynamic range and yield smaller MDE values. The principal anchor is the appropriate quantity for the bounded-null inferential question posed by this manuscript, namely whether BL PDT generates signal exceeding the LLOQ anywhere along the dynamic range over which UVB-equivalent positive control signal (67.74 to 99.71 ng/mL) is observed. Under the principal anchor, the MDE of 2.42 ng/mL exceeds the LLOQ of 1.5625 ng/mL by approximately 1.55-fold, supporting the interpretation that sub-LLOQ DNA damage cannot be excluded by the present design. Under the lower-range and at-LLOQ sensitivity anchors, the MDE falls below the LLOQ; this reflects the lower absolute variance at the lower end of the calibration curve and would warrant a more precise inferential bound (e.g., MDE ≈ 0.5 to 0.9 ng/mL) for an assay validation paper whose primary question is the reliability of the LLOQ itself. The full-dynamic-range anchor is selected as the principal MDE because the present manuscript treats the LLOQ as a validated bound and asks whether BL PDT exceeds it anywhere along the curve. SD, standard deviation; MDE, minimum detectable effect; LLOQ, lower limit of quantification.*
